# The experience of bullying among adolescents receiving mental health care: an interpretative phenomenological analysis

**DOI:** 10.1186/s13034-022-00505-7

**Published:** 2022-08-20

**Authors:** Marjorie Roques, Solène Spiers, Mayssa’ El Husseini, Didier Drieu, Dimitra Laimou, Nathalie de Kernier, Anne-Valérie Mazoyer, Fabian Guénolé

**Affiliations:** 1 Université de Bourgogne, PSY-DREPI (EA-7458), Dijon, France; 2 Pôle de Psychiatrie de l’enfant et de L’adolescent, Centre Hospitalier Le Vinatier, Bron, France; 3 Université de Picardie Jules Verne, CHSSC (EA-4289), Amiens, France; 4 Université de Rouen Normandie, CRFDP (EA-7475), Rouen, France; 5 Université Paris-Nanterre, CLIPSYD (EA-4430), Nanterre, France; 6 Université Toulouse Jean Jaurès, LCPI (EA-4591), Toulouse, France; 7 Service de Psychiatrie de l’enfant et de L’adolescent, CHU de Caen, Université Caen Normandie, 14 Avenue Clemenceau, 14033 Caen cedex 9, France

**Keywords:** Adolescent, Bullying, Harassment, Mental health services, Qualitative research

## Abstract

**Background:**

Bullying, the most prevalent form of abuse among adolescents, is associated with emotional and behavioural problems as well as psychiatric morbidity. Moreover, it has been shown that adolescents with previous mental health problems are at increased risk of being bullied and that the psychopathological repercussions of bullying are greatest among them. However, little is known about the experience of bullying in adolescents receiving treatment from mental health services. The aim of this study was to explore the subjective experience of bullying in adolescents receiving mental health care.

**Methods:**

The study was developed in the context of a French multicentre research program and employed an exploratory phenomenological approach. A purposeful sampling strategy was used to select adolescents who had experienced bullying (according to the Olweus criteria) and who were able to relate their experiences clearly. In-depth, semistructured interviews with participants were conducted; written transcriptions of these interviews were analysed using thematic analysis.

**Results:**

Twenty-one adolescents (age range: 12–17 years; 13 girls) participated in the study. The analysis indicated a three-axis structure: (1) negative emotions and violent feelings, describing adolescents’ fear, sadness, aggression against themselves, and generalized mistrust; (2) isolation and loneliness, underlining the need to take refuge within oneself and the experiences of rejection, helplessness, and secret-keeping; and (3) self and identity repercussions, including experiences of shame and lowered self-esteem, identity questions, and a vision of bullying as a life experience.

**Conclusions:**

The results of this study may have practical implications for clinicians: (1) a situation of bullying should be sought when an adolescent is seen for unexplained externalized behavioural misconduct; (2) low levels of emotional expression in a bullied adolescent may warn about associated self-harm; (3) a bullied adolescent’s tendency to hide this situation from his or her parents may reflect underlying family-related vulnerability; and (4) the phenomenological analysis showed potential particularities in the assumptive world of these adolescents and suggested that relationality may play a crucial role in their experiences. These results suggest incentives to design specific individual and group therapeutic interventions for bullied adolescents with significant levels of social withdrawal, including family support. Additional research is necessary to improve our understanding of the psychopathological and intersubjective aspects of bullying in adolescents.

## Background

Bullying is the most prevalent form of abuse among adolescents in Western countries [1, 2], with 10–20% of youth enduring it during their teenage years [3–5]. The experience of being bullied is defined as suffering repeated and intentional negative actions from an individual or group of individuals over a prolonged period, with an imbalance of power making it difficult to defend oneself on one’s own [6].

Adolescent bullying victimization mostly occurs in schools in the form of both direct and indirect actions [7]. Direct bullying includes face-to-face actions, which are usually categorized as physical (such as pushing or kicking), verbal (such as threatening or taunting), or relational (such as shaming or rejection); indirect bullying includes actions that do not require the targets to be present, such as spreading rumours or covert shaming. Cyberbullying is another common form of bullying among adolescents, which usually occurs in addition to one or more of the previous forms and appears to occur in an isolated manner only in rare cases [8, 9].

The experience of bullying can be the cause of enormous distress for adolescents [7]. Indeed, it has been shown that adolescents who have been bullied are significantly at risk of emotional problems [10] as well as both suicidal and nonsuicidal self-harm [11–15]. Moreover, several population-based studies have reported significant associations between bullying and the incidence of several mental disorders in adolescents, including anxiety and mood disorders [7, 16], posttraumatic stress disorder [17–19], and borderline personality disorder [20]. It has been shown that adolescents with a history of mental health problems, mainly including depression and borderline traits, are at increased risk of being bullied and that the psychopathological repercussions of bullying are the greatest among them [7, 21, 22]. However, very little is known regarding the experience of bullying among adolescents receiving mental health care, and there is therefore particular interest in conducting exploratory research to investigate this subject.

Among exploratory research methods, qualitative methods seem to be particularly useful in this context, as they constitute a tool of choice for understanding complex phenomena by allowing researchers to focus specifically on the views of patients, including adolescents [23, 24]. Although several qualitative studies have focused on the experience of bullying in adolescents [25–29], none have investigated this topic among adolescents receiving treatment from mental health care services. Such investigations remain-necessary to explore the emotional, cognitive, behavioural and systemic characteristics associated with bullying in clinical contexts and to contribute to the design of therapeutic interventions.

Based on a phenomenological framework, the aim of this study was to qualitatively explore the subjective experience of bullying in adolescents receiving mental health care.

## Methods

### Setting

The study was developed in the context of a French multicentre research program devoted to the psychopathology of adolescent bullying [30]. It was designed to employ a phenomenological approach with the aim of inductively exploring the representations and subjective experiences of participants [31]. Interpretative phenomenological analysis is recognized as a particularly appropriate framework for studying experiences of distress in mental health research and the meaning that subjects give to such experiences [32]. This study was conducted in accordance with the consolidated criteria for reporting qualitative research (COREQ) [33].

The study was conducted from December 2017 to November 2019 in six French hospital departments focusing on child and adolescent psychiatry (Agen, Amiens, Caen, Crosne, Castelnaudary, Vire), three school health services (Amiens, Carcassonne, Toulouse), and one foster care service (Amiens). A clinical psychologist or a psychiatrist coordinated the study at each site in concert with the principal investigator (MR).

This study was conducted in accordance with the Code of Ethics of the World Medical Association (Declaration of Helsinki, 2008) and was approved by the local committee of ethics (Comité Local d’Ethique de la Recherche en Santé du CHU de Caen; IRB#: 122017GUE). All adolescents and their parents signed informed consent forms after being provided detailed information concerning the aims and course of the study.

### Sampling

We employed a purposeful sampling strategy to select adolescents who had been victims of bullying and who were motivated to relate their experiences. Clinical coordinators at each site were careful to include participants of both sexes, with various levels of education and socioeconomic status, a variety of psychiatric diagnoses and levels of symptoms, and experiences with different forms of bullying, i.e., physical, verbal, relational, or cyber bullying. The setting of the study also allowed us to select candidates from both outpatient and inpatient care units.

The inclusion criteria were as follows: age between 12 and 17, a history of bullying according to the Olweus criteria [34], ongoing psychological care at the inclusion site, fluency in spoken French, and an interest in participating in the study. To ensure that participants had a relatively stable clinical status regarding stress-related psychopathology, the bullying was required to have ceased for at least 3 months prior to inclusion in the study. Exclusion criteria were a clinical diagnosis of schizophrenia, autism spectrum disorder, intellectual disability, or a clinical impairment that was too severe to allow for participation in the study. Mental health professionals identified potential participants in their everyday practice and, after receiving approval from the coordinators, informed the adolescents and their parents of the existence of the study and provided them with preliminary information concerning the study. If they were interested, a session was conducted with an investigator, who described the study to the adolescent and his or her parents, answered their questions, and obtained their written consent.

### Data collection

During the 3 months following this preliminary session, participants attended three meetings with a psychologist, with whom they had no prior relationship; in addition, no subsequent relationship with the psychologist was expected. These meetings included two sessions devoted to psychological tests (drawings, projective assessment) and a third session consisting of an interview regarding the experience of bullying. These verbatim interviews constitute the corpus referenced by this study.

The interviews were strictly individual and semistructured, and open-ended questions were used (Table 1). The interviews were audio-recorded with the participants’ permission. They aimed at in-depth exploration of the participants’ experiences of bullying and the meaning that they attributed to those experience, in which context participants were encouraged to openly convey their viewpoints. The interviewers were open and attentive to any new issues that participants might introduce and tried to enter the psychological and social worlds of the interviewees as much as possible; this approach was facilitated by the fact that the interviewer became familiar the participants during previous sessions. The duration of the interviews was approximately 60 min each. Recordings were transcribed verbatim, including nonverbal expressive nuances, and anonymized.

**Table 1:** Interview guide

	Area of experience	Potential questions with an adolescent
1	Bullying history, onset of bullying	How long have you been bullied? What were the circumstances?
2	Emotional reaction to bullying	What did you feel?
3	Behavioural reaction to bullying	How did you defend?
4	Perceived effectiveness of reactions	How did you cope with? What was effective?
5	Emotions after bullying has ceased	How do you feel now? What do you think of what happened?

### Analysis

Two researchers (SS and LL), who did not participate in data collection and had no theoretical or practical expertise in the field of bullying, independently analysed the corpus using the framework and methodology of interpretative phenomenological analysis [31, 32] and integrating some general methodological aspects of classical exploratory thematic analysis [35].

The analysis began by reading each complete transcript several times (familiarization [35]) prior to meticulously and iteratively annotating it line-by-line, including conceptual notes in the margins, with a focus on the experiential claims, concerns, and stances expressed by the participant [31, 32]. Initial idiographic themes were then identified, each of which categorized a number of related conceptual notes [31, 32, 35]; textual quotations illustrating the main ideas of each theme were labelled in the transcript [35]. These emerging themes were interpreted jointly by the researchers, including through the use of cross-transcript interpretations of recurrent and informative themes; they were then organized into several superordinate themes [31, 32, 35], which were extracted via associations of meanings [31, 32]. Finally, the results were synthetized to produce an ordered table of themes, with textual quotations provided for each theme [31, 32, 35].

Investigator triangulation was conducted to reinforce the validity of the results [36]: the results were regularly debated during research group meetings, which were chaired by a qualitative research expert (MEH), and discrepancies were discussed with another skilled analyst (DD) until mutual agreement was reached. The analysis concluded with several new transcripts provided no further themes, indicating theoretical saturation in the exploration of data [37].

## Results

### Participants

Interviews were proposed to 22 adolescents, 21 of whom were ultimately included in data collection (a 16-year-old girl wished to terminate her participation prior to the first interview without providing a reason). The basic demographic and clinical characteristics of the participants are summarized in Table 2**.** All participants experienced physical, verbal and relational bullying; six participants had also been cyber-bullied. In accordance with the sampling strategy, participants were of both sexes and displayed heterogeneity regarding age (from 12 to 17 years), socioeconomic status and level of education (ranging from low to high and from 7 to 11^th^ grade, respectively) as well as the duration of the bullying they experienced (from 2 months to 10 years). Both outpatients and inpatients were included, with a variety of psychiatric diagnoses and levels of symptoms.

**Table 2:** Basic demographic and clinical characteristics of participants

	Age	Sex	Type(s) of bullying	Duration of bullying	Psychiatric Diagnosis	Clinical severity	Care setting	School level	Socioeconomic status
A1	14	M	Physical Relational Verbal	2 months	Disinhibited attachment disorder Nonorganic enuresis	Moderate	Outpatient	8th	Low
A2	12	F	Cyber Physical Relational Verbal	2 months	Nonorganic insomnia	Mild	Outpatient	7th	Low
A3	15	M	Physical Relational Verbal	2 years and 6 months	Social anxiety	Mild	Inpatient	BTEC1	Middle
A4	16	M	Physical Relational Verbal	1 year	Somatization disorder Separation anxiety disorder	Severe	Outpatient	NVQ1	Middle
A5	17	F	Physical Relational Verbal	4 years	Depressive episode Post-traumatic stress disorder	Severe	Inpatient	11th	High
A6	13	F	Physical Relational Verbal	1 year and 6 months	None	Low	Outpatient	7th	High
A7	16	F	Physical Relational Verbal	1 year	Post-traumatic stress disorder	Moderate	Outpatient	11th	High
A8	14	F	Cyber Physical Relational Verbal	2 years	Recurrent depressive disorder Post-traumatic stress disorder	Moderate	Inpatient	9th	Middle
A9	14	M	Cyber Physical Relational Verbal	1 year	Depressive episode Post-traumatic stress disorder	Severe	Outpatient	7th	Middle
A10	16	M	Physical Relational Verbal	2 years	Adjustment disorder	Severe	Inpatient	9th	Middle
A11	16	F	Physical Relational Verbal	5 months	Reactive attachment disorder	Mild	Inpatient	10th	Middle
A12	12	F	Physical Relational Verbal	2 years	Nonorganic insomnia	Low	Outpatient	7th	Middle
A13	17	F	Physical Relational Verbal	3 years	Post-traumatic stress disorder	Mild	Outpatient	11th	Low
A14	16	F	Cyber Physical Relational Verbal	10 years	Post-traumatic stress disorder	Mild	Outpatient	10th	Middle
A15	13	M	Physical Relational Verbal	7 years	Agoraphobia Social anxiety Generalized anxiety disorder	Severe	Outpatient	8th	Middle
A16	17	F	Physical Relational Verbal	5 months	Depressive episode	Mild	Inpatient	NVQ2	Low
A17	16	F	Physical Relational Verbal	4 years	Depressive episode	Severe	Outpatient	9th	Middle
A18	12	M	Cyber Physical Relational Verbal	3 months	Generalized anxiety disorder	Mild	Outpatient	7th	Low
A19	16	M	Cyber Physical Relational Verbal	4 years	Conduct disorder	Severe	Outpatient	BTEC1	Low
A20	16	F	Physical Relational Verbal	6 years	Post-traumatic-stress disorder Reactive attachment disorder	Moderate	Outpatient	9th	Low
A21	17	F	Physical Relational Verbal	1 year	Depressive episode Post-traumatic stress disorder	Moderate	Outpatient	BTEC2	Middle

### Thematic analysis

**Fig. 1:**
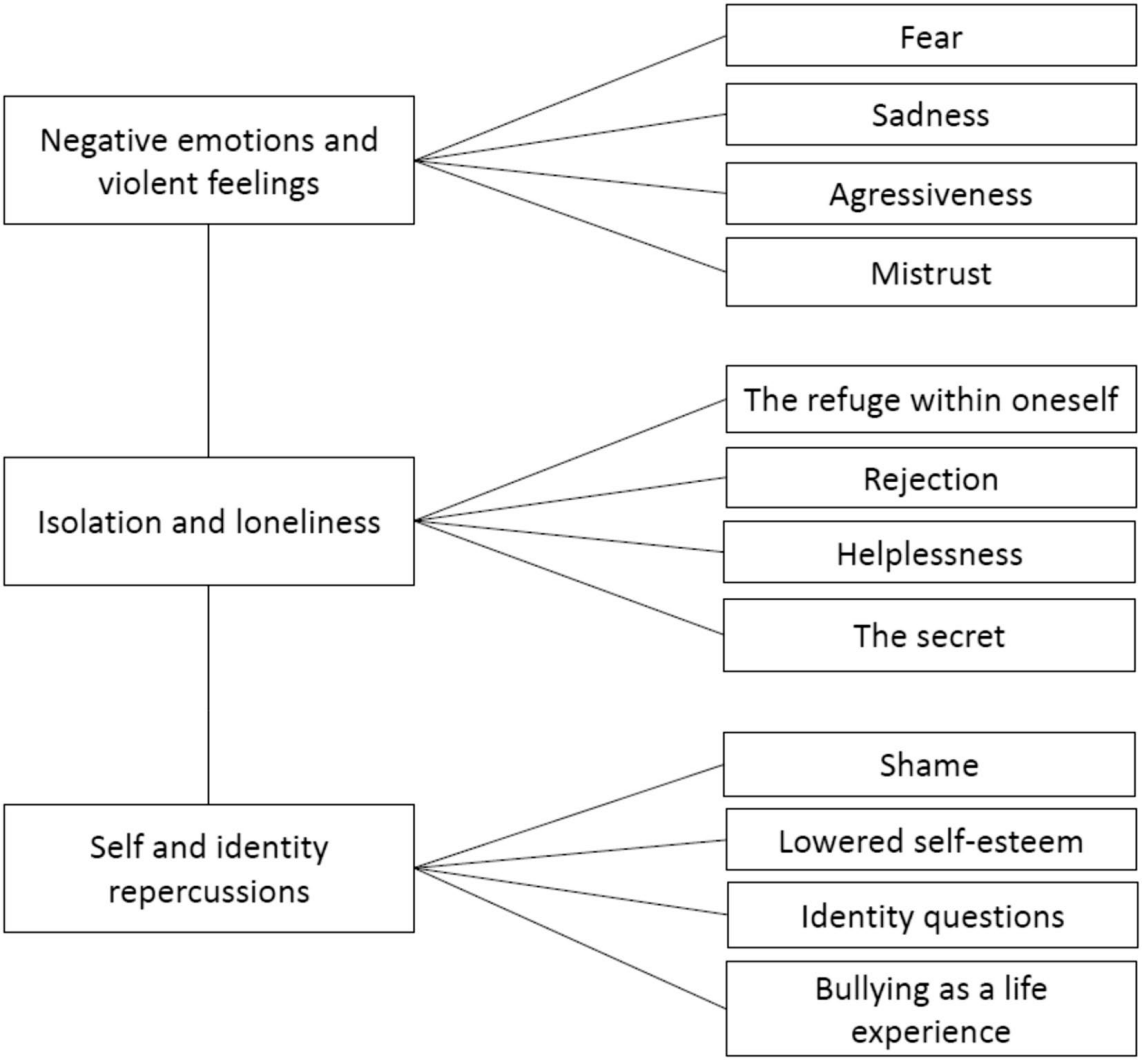
Structure of the thematic analysis

The phenomenological thematic analysis of the interviews enabled us to identify 12 themes, which were organized into 3 superordinate themes (Fig. 1): (1) negative emotions and violent feelings; (2) isolation and loneliness; and (3) self and identity repercussions. These themes are presented below alongside exemplary quotations; all meaningful quotations are listed in Table 3, translated into English.

**Table 3:** List of quotations

1. Negative emotions and violent feelings	
	1.1. Fear Q1. Huh, I was in the stairwell and I could see, well I see, I mean I could see them … and I wasn’t okay [embarrassed laugh] … yeah … because, well I mean there’s always some stress. Yes, especially after all that, yeah Q2. On my way out of school […] I was always scared that some moron would be waiting for me around the corner to kill me Q3. I didn’t necessarily have the guts to defend myself at the time, I didn’t really dare, I, I was a bit scared of the repercussions that would follow Q4. I would tremble, I wasn’t well, […] I would vomit
	1.2. Sadness Q5. I would cry for nothing Q6. Yeah, you know, the urge to cry immediately Q7. At the slightest thing, you know, I can cry but I don’t do it in front of everyone Q8. I was destroyed, well, I couldn’t stop crying, it was really… Q9. I couldn’t work anymore, I couldn’t concentrate on my work, I cried all the time in the evenings, I didn’t feel like doing that
	1.3. Aggressiveness Q3. I didn’t necessarily have the guts to defend myself at the time, I didn’t really dare, I, I was a bit scared of the repercussions that would follow Q10. Hate, obviously Q11. A little bit of hate I admit, they were aware of what they were doing, they were not in a trance you know, nor in an altered state. No, no, they took pleasure in hurting me, in seeing me suffer, so I feel no compassion for them or any need to forgive them Q12. I know that this girl is manipulative, that doesn’t surprise me about her now that I’ve seen the side of her that can be so nasty Q13. Yeah… well actually I come off as…someone nice but in reality deep within me if I’m pushed… If they don’t push me I’ll be nice, if they do well…I’ll take on a different form Q14. The desire to go after those people, to answer back but I, I didn’t have the guts to do it Q15. All the hatred that you feel for yourself, so you feel like mutilating yourself, hurting yourself… and at the same time feeling good, to feel alive in quotes Q16. Uh, in the middle of the 7th grade I started to cut myself. And then, well, later it was my brother who discovered it at the end of the school year in the 9th grade. [Interviewer: That lasted for a year and a half?] Yes, and it was two months before the end of the year Q17. The end of junior high, early high school, that’s when I didn’t hesitate to cut myself Q18. [Interviewer: Did you go further than the cuttings?] Yes, […] I was going to try. [Interviewer: And what stopped you?] Well, my brother Q19. Yeah, no I got tired of it and I wanted to end my life and then um… Q20. [Interviewer: You had dark thoughts?] Yes Q21. [Interviewer: Some kind of plan?] Uh, I had prepared a stock of drugs um for the day when, when I would have wanted to Q22. I’m at, I attempted to commit suicide… I attempted to commit suicide and it was complicated because I wanted to do it but still I didn’t have … have the… have the balls in quotes to do it… uh
	1.4. Mistrust Q12. I know that this girl is manipulative, that doesn’t surprise me about her now that I’ve seen the side of her that can be so nasty Q23. And actually, there were several boys who wanted to go out with me and I didn’t want to, and I was in love with a boy who wasn’t in love with me. And so, um… we would send each other messages but in fact he made me believe that he too was in love. So, in fact I got caught up in his game and … I’m thinking, maybe something can happen between us. And in the end, it turned out that that wasn’t it, and… uh… and he… he set up all the boys in the class against me Q24. She had told everyone that I had led her to suicide, etc. So well the people believed it … they left me alone all the time they didn’t want to talk to me anymore Q25. It was mostly a group of people who were manipulated by the person I had dated, um… really rebelling against me, it really hurt because they tried to get everyone against me. I felt really lonely and that’s what hurt me the most Q26. I can see that they talk behind my back, I’ve got people who come and tell me, I can see very well that some things are done so it’s…it’s always annoying, it’s always there… Q27. It began in middle school; I was friends with a group of girls but then I had a disagreement with one of them. I started dating a boy and she didn’t like it because I was no longer with her as much. She started spreading rumors about me Q28. Yes, I was becoming almost paranoid, suspicious of everything, because of being betrayed and deceived by people who I thought were my friends Q29. It’s true that it was hard because she was my best friend … Q30. Overnight she was my ex-best friend, I didn’t understand it, she turned on me and started insulting me [and] all that … Q31. There’s my ex-boyfriend who, now that I’m longer with him, bullies me too Q32. It started because I was with him, and then he turned his back on me Q33. That we were a group of girls and… overnight, they told me … well it was toward the end of the 7th grade; a group of girls would say to me “yeah, you dress badly" … well,” you’re stupid”, “you don’t know how to do anything”, so they would tell me that every day Q34. He kind of got all the boys in the class against me Q35. Everyone’s mad at me, taking turns, I don’t get it Q36. When people, teenagers have decided that we are the target in quotes, well that’s it, everyone is against us, and so it’s a very difficult experience because after that it’s not just necessarily in college, it also spreads to social networks Q37. Even when I meet people I don’t know, they push me around and know my name Q38. It was really almost the entire high school, well the entire middle school Q39. It went so far that when I would hear someone laughing, I would think they were laughing about me Q40. I was suspicious of others Q41. I’d say yes and it’s normal that today I’m quite suspicious, at least more than before, always a little apprehensive about who people are, their personality, what they really think. I’m relatively good at identifying people, their profiles in quotes Q42. I don’t trust many people; I even find it difficult within my family. So um, I find it hard to trust people Q43. I knew for a fact who had done it and everyone was in cahoots with that Q44. Everyone wanted to go out with me to prove to everyone else that I was an easy girl
2. Isolation and loneliness	
	2.1. The refuge within oneself Q45. I didn’t take this perception of others well, I would lower my head as I walked, with timid, withdrawn positions. That lasted a long time, you never forget Q46. I would walk fast and I was always in a hurry to get home Q47. I didn’t come to high school for a few days, I could no longer set foot here, I couldn’t
	2.2. Rejection Q48. I don’t feel accepted, really not at all Q49. I’m not taken seriously Q50. I would try to fit in but no one accepted me. It’s hard Q51. So, I eat lunch with friends, sometimes they say, “well no, you’re not supposed to be here, we don’t want you and everything …”. When at times I talk to people with whom I get along and there’s this person, well who bugs me, he tells me “Oh shut up, get lost”, well things like that, well, repeated, repeated, repeated, that, that, it’s hard to experience, it’s rather complicated Q52. People rejected me a lot, it was a lot of rumors about my case, I was really not accepted and so I actually suffered quite a bit Q53. I mostly feel that, you know, that they use that to hurt me as much as possible then exclude me from everything well as soon as, as soon as there is something with everyone, they tell me “‘get lost, you’ve got nothing to do here”!
	2.3. Helplessness Q25. It was mostly a group of people who were manipulated by the person I had dated, um… really rebelling against me, it really hurt because they tried to get everyone against me. I felt really lonely and that’s what hurt me the most Q48. I don’t feel accepted, really not at all Q54. They left me alone all the time, they didn’t want to talk to me anymore … Q55. They left me alone, I was really on my own Q56. Isolated, really very isolated Q57. At times it really made me lose my self-esteem you know… eventually, every day, well a criticism now and then, so as long as it’s constructive it’s no problem, but when it’s not constructive and it’s repeated every day, well eventually you begin to have doubts […] when you’re… well especially when you’re alone I think when you have no one around you, even if your parents are there you don’t necessarily have the guts to tell them and … so you dwell on it, you dwell on it and ask yourself many questions and … and you question yourself a lot Q58. Even though I was surrounded by lots of people I felt lonely. I felt like no one understood me and no one could help me Q59. Everyone was acting like it was normal, normal there’s a girl getting pushed around in the hallway … nobody cares … Q60. You know that’s what I’m saying, when I fought with Vanessa, what did my supervisor say, she said ‘yes, well, you only have to make a wig with it’ or in the 9th grade my finger got caught in the door um… yeah I broke my little finger, well just the little bit here, I went to see my supervisor, she laughed in my face… so I went straight to the principal’s office and then I showed him my finger, from there the emergency services came in, well that’s it, no, no one gave a damn about me, that’s good it’s her life not ours Q61. I felt like I was also bothering them at the police station, the impression that I wasn’t necessarily being listened to … hard to be taken seriously. The impression that when I spoke to them about that, it was not that bad because there weren’t that many calls, that it wasn’t that bad
	2.4. The secret Q57. At times it really made me lose my self-esteem you know… eventually, every day, well a criticism now and then, so as long as it’s constructive it’s no problem, but when it’s not constructive and it’s repeated every day, well eventually you begin to have doubts […] when you’re… well especially when you’re alone I think when you have no one around you, even if your parents are there you don’t necessarily have the guts to tell them and … so you dwell on it, you dwell on it and ask yourself many questions and … and you question yourself a lot Q62. I would say nothing, I would keep silent… without saying anything… Q63. I hadn’t told anyone Q64. We would say nothing Q65. I didn’t talk about it because I was ashamed Q66. I couldn’t talk about it Q67. I would say nothing but I would cry Q68. I kept everything inside Q69. I had already told her about it because you know I came home crying, I was angry, I was hitting everything … well especially the bed but… then well there was nothing. Then this year I hadn’t told her about this year, I really started talking to her about it you know… when it really started getting unbearable and she would see that I was crying but well… It was mainly my drum teacher who advised me to talk to my mom, who told me not to stay that way Q70. My parents, uh, I didn’t tell them about it Q71. I didn’t really want to talk to my parents Q72. Well, I would pretend that it was fine… but in my head, it wasn’t Q73. I had bruises in the eighth grade, one of the classmates I sat next to would punch me in the thigh because I supposedly took too much space at the table. When my mother would ask where all the bruises came from, I would lie and say that I had bumped into something Q74. It’s true that I took a long time to talk to her, not because, I don’t know, well I was withdrawn it’s true that, about everything about many other things. I used to be quite shy, and um, I would keep a lot to myself and I would say that’s what really destroyed me Q75. I waited until the ninth grade to talk about it, the day two guys, twin brothers, tried to grab me on the way out [of school]and my dad caught them. I told my parents everything after that, 6 months before the middle high school certificate and overnight, I was no longer bullied. I think it scared them; they saw the limits. But I was even more alone after that, I passed for a sucker Q76. It’s not my goal, I don’t want to get into trouble and get my mom into trouble because of kids’ reactions in quotes Q77. Talking about it always evokes heartbreaking memories, naturally, it’s not something one forgets, but it also helps other victims to talk about it while they were hiding themselves in silence. It’s important to talk about it. Honestly, it helped me, so I’m doing everything I can to get others to talk about it. And writing also helped me get through it Q78. I actually talked about it easily, I’d been going through that for several years… I figured it was pointless to hide, it was like carrying a weight on my own so I thought I might as well talk about it because it allows to, well, at least so people know and it’s important because it involved people from high school Q79. I think I’ve changed since I’ve been here, even though it will only be a week tomorrow, but there’s a change, I see it myself. Even as I speak to people, I am able to open up, like to you or to psychologists and doctors. I think it’s cool Q80. I didn’t know that I could talk to people I could trust, psychologists and all that. Now it’s something I know so if one day I’m not doing too great, I think I’d make an appointment with these people. And it’s something that’s good to talk about. Something I didn’t dare do before but it’s true that talking, it does a world of good. We feel liberated actually we get all our hatred out, all our anger yeah it feels good
3. Self and identity repercussions	
	3.1. Shame Q65. I didn’t talk about it because I was ashamed Q81. Actually, I was too ashamed Q82. Uh. Then as soon as I had something that was not that normal, I would feel ashamed I … I would think to myself ‘why did I do that, why did I wear this’ people would feel it, they would laugh at me Q83. I didn’t dare take my clothes off because I was ashamed of my body and the others would laugh at me Q84. I would feel ashamed… Why me, it hurts me and not the others?
	3.2. Lowered self-esteem Q57. At times it really made me lose my self-esteem you know… eventually, every day, well a criticism now and then, so as long as it’s constructive it’s no problem, but when it’s not constructive and it’s repeated every day, well eventually you begin to have doubts […] when you’re… well especially when you’re alone I think when you have no one around you, even if your parents are there you don’t necessarily have the guts to tell them and … so you dwell on it, you dwell on it and ask yourself many questions and … and you question yourself a lot Q85. I didn’t have any confidence in myself at all Q86. I had lost all confidence in myself. Really, I was trying to find myself… Q87. In the end everything they would tell me, I would think “ah maybe they’re right” and suddenly I couldn’t look at myself in the mirror anymore […]. And I don’t know I found myself disgusting, and I would think, “they’re actually right” Q88. When I would try on a top, as soon as I would buy a top I would think, “Will people like it, will people like it?” Did I like it? No I didn’t ask myself that question Q89. I’ve never been self-confident so after, let’s say that didn’t help make me more confident Q90. After, uh, one tries to restore self-confidence little by little
	3.3. Identity questions Q84. I would feel ashamed… Why me, it hurts me and not the others? Q91. It started I don’t know, well maybe because I was weaker or more sensitive, well also I’m a little different… well I’m sturdier, well, more sturdy in the sense of stocky, I’m more … well, I’m not really like everyone else Q92. I have a slightly different way of reacting, I can… well I’m more sensitive, at the slightest thing well I can cry but I don’t do it in front of everyone, then I’m a bit violent, well not violent, I can talk back to people and they don’t like that Q93. I was different, that’s all Q94. [Interviewer: Why do you feel weaker than others?] Mentality then … height. And then um in the way I express myself when I… they don’t take me seriously Q95. Well… they would avoid me… because I was different … Q96. I’m very small, I have a problem with my leg so I’ve always been mocked Q97. Because I’m small Q98. Well, I didn’t dress like them. […] Well, I wore… I didn’t wear leading clothing trademarks; I didn’t have any branded shoes so you have to avoid people like that… Q99. I’ve always wanted it, to be different, not to look like everyone else Q100. I would try to fit in, but no one accepted me. It’s hard. I wasn’t easily influenced, I didn’t try to drink or smoke to be like them, so they felt I was different. I didn’t want to follow this mold imposed by everyone there … Q101. I wanted to always reflect the image of what they wanted Q102. Not looking like everyone else, it really brought me problems
	3.4. Bullying as a life experience Q41. I’d say yes and it’s normal that today I’m quite suspicious, at least more than before, always a little apprehensive about who people are, their personality, what they really think. I’m relatively good at identifying people, their profiles in quotes Q45. I didn’t take this perception of others well, I would lower my head as I walked, with timid, withdrawn positions. That lasted a long time, you never forget Q103. What has changed today is that I understand that the person in front of me is just like me, that he is not superior, that I am not superior to her and that everything must be based on some form of dialogue, and well at the time I didn’t understand it at all Q104. At the same time, it’s a good experience, well in quotes because it makes us grow, it makes us become aware of things and that … in fact it’s possible that without that I would not be the person that I am today, not as mature not as um … thoughtful. So, it made me move forward, it helped me like it destroyed me. […] The past helps us move forward, not everyone, but I know that the past helped me move forward, made me mature, made me realize things that I had not … Q105. No, I would even say that it strengthened me. I built myself around that. That’s what I was telling you, it began very early and it was more or less nasty each time. […] I may have passed this test; it may help me to understand the future tests that I might encounter in my future life

### Negative emotions and violent feelings

Fear. Participants reported having felt fear in relation to bullying. This fear could become manifest in the presence of bullies:

“*Huh, I was in the stairwell and I could see, well I see, I mean I could see them... and I wasn’t okay* [embarrassed laugh] *… yeah … because, well I mean there’s always some stress. Yes, especially after all that, yeah.*”

It could also take the form of an anticipatory fear on a daily basis. This variety included, for example, the fear of being ambushed or, in a more diffuse way, anxious ruminations regarding possible and still ignored negative consequences of bullying:

*“On my way out of school* […] *I was always scared that some moron would be waiting for me around the corner to kill me.”*

“*I didn’t necessarily have the guts to defend myself at the time, I didn’t really dare, I, I was a bit scared of the repercussions that would follow*.”

Anxiety could have significant physical manifestations:

“*I would tremble, I wasn’t well,* […] *I would vomit*.”

*Sadness*. Another negative emotion felt in connection with bullying was sadness, which become manifest in the form of very easy and frequent crying:

“*I would cry for nothing*.”

“*Yeah, you know, the urge to cry immediately*.”

Tearfulness could occur preferentially at home in a lonely and concealed way:

“*At the slightest thing, you know, I can cry, but I don’t do it in front of everyone*.”

A participant noted that her sadness reflected a feeling of collapse. Feelings of being depressed could be a source of difficulty with completing homework:

“*I was destroyed, well, I couldn’t stop crying, it was really...* […] *I couldn’t work anymore, I couldn’t concentrate on my work, I cried all the time in the evenings, I didn’t feel like doing that*.”

Aggressiveness. Participants expressed that they felt hatred towards those who bullied them. As they described it, this hatred was experienced in response to the violence they suffered from their bullies, whom they felt had exhibited voluntary nastiness or even sadistic cruelty:

“*A little bit of hate I admit, they were aware of what they were doing, they were not in a trance you know, nor in an altered state. No, no, they took pleasure in hurting me, in seeing me suffer, so I feel no compassion for them or any need to forgive them*.”

However, the hatred and violence participants felt could be expressed in euphemistic and rather repressed terms; they did not act on these emotions due to the inhibitions caused by fear:

“*The desire to go after those people, to answer back but I, I didn’t have the guts to do it*.”

A girl described that she engaged in self-harm as a consequence of directing her hatred against herself and that she experienced more vitality from doing so:

“*All the hatred that you feel for yourself, so you feel like mutilating yourself, hurting yourself... and at the same time feeling good, to feel alive in quotes*.”

Other participants reported that they had self-injured. Suicidal ideations and conduct were also reported.

*Mistrust*. The feeling associated with realizing the wickedness of bullies was described as uncovering something previously hidden. Participants reported impressions of having been deceived by manipulative persons. They described recurrent slanders and false rumours about them, which they viewed as secret manipulations. These situations could be experienced as a form of treason, as they had occasionally been caused by friends; the changes in attitude exhibited by these individuals may have been viewed as a sudden turnaround:

“*Yes, I was becoming almost paranoid, suspicious of everything, because of being betrayed and deceived by people who I thought were my friends*.”

“*Overnight she was my ex-best friend, I didn’t understand it, she turned on me and started insulting me [and] all that* …”

Some participants perceived an escalation of social hostility starting from one malicious person and spreading throughout the whole group:

“*He kind of got all the boys in the class against me*.”

“*Everyone’s mad at me, taking turns, I don’t get it*.”

This situation caused lived experiences of perplexity and could go beyond the circle of familiar individuals:

“*Even when I meet people I don’t know, they push me around and know my name*.”

“*When people, teenagers have decided that we are the target in quotes, well that’s it, everyone is against us, and so it’s a very difficult experience because after that it’s not just necessarily in college, it also spreads to social networks*.”

This impression of latent and diffuse social hostility was described as a cause of hypervigilance. The source of mistrust, which could reach intense levels and extend to all social relationships, was also described:

“*I’d say yes and it’s normal that today I’m quite suspicious, at least more than before, always a little apprehensive about who people are, their personality, what they really think. I’m relatively good at identifying people, their profiles in quotes*.”

“*I don’t trust many people; I even find it difficult within my family. So um, I find it hard to trust people*.”

### Isolation and loneliness

*Taking refuge within oneself*. It was noted that fear had been a source of inhibition and introversion and caused a reaction of flight from the outside world:

“*I didn’t take this perception of others well, I would lower my head as I walked, with timid, withdrawn positions. That lasted a long time, you never forget*.”

Defensive withdrawal was reported alongside thoughts and behaviours associated with diversion, which were mainly solitary.

*Rejection*. Painful feelings of not having been socially accepted or taken into consideration by peers were reported by participants, despite their reported attempts to integrate themselves into the social group. Additionally, participants described being repeatedly rejected because of negative rumours or even the impression that this social rejection was actually aimed at making them suffer from isolation:

“*So, I eat lunch with friends, sometimes they say, “well no, you’re not supposed to be here, we don’t want you and everything ...”. When at times I talk to people with whom I get along and there’s this person, well who bugs me, he tells me “Oh shut up, get lost”, well things like that, well, repeated, repeated, repeated, that, that, it’s hard to experience, it’s rather complicated*.”

“*I mostly feel that, you know, that they use that to hurt me as much as possible then exclude me from everything well as soon as, as soon as there is something with everyone, they tell me “‘get lost, you’ve got nothing to do here*”!”

*Helplessness*. Participants reported feeling lonely and isolated within their peer group. Loneliness has been described as the source of rumination, a feeling of powerlessness, and the inability to obtain help:

“*At times it really made me lose my self-esteem you know... eventually, every day, well a criticism now and then, so as long as it’s constructive it’s no problem, but when it’s not constructive and it’s repeated every day, well eventually you begin to have doubts [...] when you’re... well especially when you’re alone I think when you have no one around you, even if your parents are there you don’t necessarily have the guts to tell them and ... so you dwell on it, you dwell on it and ask yourself many questions and ... and you question yourself a lot*.”

It seems that these negative thoughts were linked to a feeling of being misunderstood and indifference from others, including adults should have acted and may have trivialized the situation:

“*Even though I was surrounded by lots of people I felt lonely. I felt like no one understood me and no one could help me.*”

“*Everyone was acting like it was normal, normal there’s a girl getting pushed around in the hallway ... nobody cares ...*”

*The secret*. Participants noted that they had remained silent about the bullying they suffered and did not have the ability to tell anyone about it:

“*I would say nothing, I would keep silent... without saying anything*...”

“*I couldn’t talk about it*.”

Some adolescents, however, described having expressed their angst indirectly:

“*I would say nothing but I would cry*.”

“*I had already told her about it because you know I came home crying, I was angry, I was hitting everything ... well especially the bed but... then well there was nothing. Then this year I hadn’t told her about this year, I really started talking to her about it you know... when it really started getting unbearable and she would see that I was crying but well ...*”

This reluctance to discuss bullying also pertained to parents, which may have resulted in dissimulation and lies:

“*My parents, uh, I didn’t tell them about it*.”

“*I didn’t really want to talk to my parents*.”

“*I had bruises in the eighth grade, one of the classmates I sat next to would punch me in the thigh because I supposedly took too much space at the table. When my mother would ask where all the bruises came from, I would lie and say that I had bumped into something*.”

Participants said they waited a long time to tell their parents that they were being bullied, a silence which occasionally persisted until this situation could no longer be ignored:

“*It’s true that I took a long time to talk to her, not because, I don’t know, well I was withdrawn it’s true that, about everything about many other things. I used to be quite shy, and um, I would keep a lot to myself and I would say that’s what really destroyed me*.”

“*I waited until the ninth grade to talk about it, the day two guys, twin brothers, tried to grab me on the way out* [of school] *and my dad caught them. I told my parents everything after that, 6 months before the middle high school certificate and overnight, I was no longer bullied. I think it scared them; they saw the limits. But I was even more alone after that, I passed for a sucker*.”

This silence towards parents was described as a way of avoiding causing difficulties for them:

“*It’s not my goal, I don’t want to get into trouble and get my mom into trouble because of kids’ reactions in quotes*.”

In some cases, a trusted person outside the family successfully prompted the adolescent to reveal this secret to his or her parents:

“*It was mainly my drum teacher who advised me to talk to my mom, who told me not to stay that way*.”

Some participants explained that breaking their silence about the bullying caused them to feel better:

“*I actually talked about it easily, I’d been going through that for several years... I figured it was pointless to hide, it was like carrying a weight on my own so I thought I might as well talk about it because it allows to, well, at least so people know and it’s important because it involved people from high school.*”

“*I didn’t know that I could talk to people I could trust, psychologists and all that. Now it’s something I know so if one day I’m not doing too great, I think I’d make an appointment with these people. And it’s something that’s good to talk about. Something I didn’t dare do before but it’s true that talking, it does a world of good. We feel liberated actually we get all our hatred out, all our anger yeah it feels good.*”

### Self and identity repercussions

*Shame*. Adolescents expressed having felt ashamed of themselves. This feeling of shame could come from recurrent mockery on the part of peers – regarding their physical appearance, for example, their clothes or bodily appearance, their behaviour, and more generally any characteristic that was perceived as a difference:

“*Uh. Then as soon as I had something that was not that normal, I would feel ashamed I ... I would think to myself ‘why did I do that, why did I wear this’ people would feel it, they would laugh at me*.”

“*I didn’t dare take my clothes off because I was ashamed of my body and the others would laugh at me*.”

A girl also described being ashamed of feeling emotionally vulnerable compared to her peers:

“*I would feel ashamed... Why me, it hurts me and not the others*?”

*Lowered self-esteem*. Participants noted that they lost confidence in themselves. This lack of confidence has been linked with the experience of repeated mockery over time. Their self-esteem was described as having become increasingly contingent on the gazes and opinion of others, which may have led to self-doubt or even self-loathing:

“*In the end everything they would tell me, I would think “ah maybe they’re right” and suddenly I couldn’t look at myself in the mirror anymore* [...]*. And I don’t know I found myself disgusting, and I would think, “they’re actually right*”.”

“*When I would try on a top, as soon as I would buy a top I would think, “Will people like it, will people like it?” Did I like it? No I didn’t ask myself that question*.”

Self-esteem was described as having previously been fragile, and it could be restored gradually after the bullying ceased.

*Identity questions*. Adolescents noted that they felt different from their peers. This perceived difference could be physical, related to their bodies or clothing, or psychological or behavioural:

“*It started I don’t know, well maybe because I was weaker or more sensitive, well also I’m a little different ... well I’m sturdier, well, more sturdy in the sense of stocky, I’m more ... well, I’m not really like everyone else*.”

“*Well, I didn’t dress like them.* […] *Well, I wore... I didn’t wear leading clothing trademarks; I didn’t have any branded shoes so you have to avoid people like that* ...”

“*I have a slightly different way of reacting, I can... well I’m more sensitive, at the slightest thing well I can cry but I don’t do it in front of everyone, then I’m a bit violent, well not violent, I can talk back to people and they don’t like that*.”

While bodily and psychological differences were described as being suffered passively, differences from peers in terms of clothing or social behaviour could be considered deliberate:

“*I’ve always wanted it, to be different, not to look like everyone else*.”

“*I would try to fit in, but no one accepted me. It’s hard. I wasn’t easily influenced, I didn’t try to drink or smoke to be like them, so they felt I was different. I didn’t want to follow this mould imposed by everyone there* ...”

A girl felt as if she were psychologically vulnerable compared to her peers and described questioning the reasons for this supposed vulnerability; similarly, a boy described wanting to conform to the social identity assigned to him by his peers:

“*I would feel ashamed... Why me, it hurts me and not the others*?”

“*I wanted to always reflect the image of what they wanted*.”

All of the types of differences mentioned by participants were described as contributing to the situation of being bullied.

*Bullying as a life experience*. The period of bullying may have left some particularly vivid memories. A boy noted that this difficult experience improved his ability to analyse the behaviour of others:

“*I’d say yes and it’s normal that today I’m quite suspicious, at least more than before, always a little apprehensive about who people are, their personality, what they really think. I’m relatively good at identifying people, their profiles in quotes*.”

A girl explained that this experience changed her relationships with her peers, which shifted from submission or the pursuit of dominance to a more symmetrical and reciprocal form:

“*What has changed today is that I understand that the person in front of me is just like me, that he is not superior, that I am not superior to her and that everything must be based on some form of dialogue, and well at the time I didn’t understand it at all*.”

Another girl noted that, despite the suffering that the bullying had caused for her, this experience had a psychologically maturing effect, i.e., it had developed her thinking skills:

“*At the same time, it’s a good experience, well in quotes because it makes us grow, it makes us become aware of things and that ... in fact it’s possible that without that I would not be the person that I am today, not as mature not as um ... thoughtful. So, it made me move forward, it helped me like it destroyed me*. […] *The past helps us move forward, not everyone, but I know that the past helped me move forward, made me mature, made me realize things that I had not* ...”

Finally, a boy felt that he had gained psychological force from the ordeal of having been bullied, which he believed he could use to cope with adversity in the future:

“*No, I would even say that it strengthened me. I built myself around that. That’s what I was telling you, it began very early and it was more or less nasty each time*. […] *I may have passed this test; it may help me to understand the future tests that I might encounter in my future life*.”

## Discussion

Although the high frequency of a history of bullying among adolescents who undergo consultations regarding their mental health is well known [16, 38], no qualitative study has explored this specific context, and to our knowledge, this study is the first to investigate the experience of bullying in adolescents suffering from mental health distress using individual, in-depth interviews.

The study highlights elements that may contribute to extending our knowledge of the experiences already described in nonspecific adolescent contexts. This contribution is particularly noteworthy regarding the extent of the emotional impact of bullying [25, 27–29] and its lack of disclosure by adolescents [26].

The results of this study show that, although feelings of anger and hatred towards bullies are felt intensely, they may have a mitigated expression in bullied adolescents, and this effect seems to be rendered indirect by fear. The analysed corpus also indicates the possibility of a reversal of such aggression to the point that it can be directed against oneself, thus appearing as a form of regulation of negative emotions, as has been described in a general conception of self-harm in adolescents [39–41]. Suicidal behaviour and self-injury, two significantly frequent clinical manifestations in adolescents who have been bullied [12, 42], could thus be associated with adolescents’ inability to express certain negative emotions. Our analyses also showed that adolescents could express their angst indirectly, in particular via externalized behavioural misconduct, because they did not dare to reveal the situations they were facing directly. Externalized behavioural misconduct has been shown to be associated with being bullied in adolescents [43], and this connection may be explained by our qualitative results.

This latter point is connected to the fact that adolescents did not disclose the bullying they faced to their parents. A previous qualitative study of the reasons underlying adolescents’ failure to disclose such bullying suggested an association with several themes: the ubiquitous nature of bullying, a sense of helplessness, concerns regarding inappropriate adult action, self-reliance, shame, parental omniscience, and a different definition of bullying than that used by adults [26]. Our results suggest that the desire to avoid causing difficulties for parents constitutes an additional psychological factor for adolescent’s failure to disclose bullying, which could thus be more or less specific to adolescents receiving mental health care and may represent the reason why bullying can be revealed to a trusted person outside the family.

A number of the experiences described by adolescents in this study include the phenomenon of dissimulation: bullying is silenced, lies are invented to hide this situation, and the expression of personal suffering or that of emotions in general is reduced, encrypted, or solitary, as are self-harming behaviours. Dissimulation to others seems to respond to a “hermeneutics of suspicion” [44]: a perceived hostility associated with the outside world is experienced as a sudden revelation of evil [45], which was hitherto unapparent due to the duplicity of peers. Everything seems to occur as if caused by a persistence and proliferating conspiracy, leading to the resulting feelings of betrayal. These more or less persecutory feelings cause the individual to anticipate further disclosures of evil, and the individual’s social group thus becomes intensely mistrusted. The individual’s loss of the feeling of belonging to the group following rejection and/or withdrawal tends to modify the psychological balance that depends on these interactions, even in adolescents who take a peripheral position in their peer groups [46]. The social isolation that results—even if it may initially constitute a form of protection—thus seems to be a factor leading to psychological imbalance and may result in the reinforcement of the common adolescent tendency towards distrust [47]. This process could represent a vicious cycle for some adolescents, especially those who lack compensatory parental and family support [48].

Though only a minority of adolescents had a clinical diagnosis of post-traumatic stress disorder, this ensemble evokes the phenomenology of traumatic experience, described by Stolorow as typically including an intersubjective context in which mental suffering can neither be expressed to nor understood by an alter ego [49]. According to this phenomenological approach, relational isolation following highly adverse events leads to a collapse of the world’s “public interpretedness”, that is, the phenomenon of “no-longer-being-at-home” in one’s environment, which thus loses the tranquilized familiarity that is associated with everyday social exchange. The resulting feelings of perplexity and uncanniness, which can readily be associated with traumatic anxiety, are believed to be connected to this phenomenological complex [50]. The possibility of establishing a connection with someone who shows him- or herself to be capable of understanding emotional experiences related to bullying could account for the fact that the adolescents in question, who were previously unable to tell anyone about this situation, described the act of breaking this silence as causing them to feel better [50, 51].

A contrast emerged in the results of this study between the vividness of the negative memories associated with bullying and the rather positive experiences of the subsequent psychological consequences of this situation, which were described in terms of the maturation of social understanding and expectations as well as assertiveness. Such optimistic self-evaluations also stand in contrast with adolescents’ expressions of shame at the possibility of appearing emotionally vulnerable. We thus hypothesize that adolescents can work through painful emotions by shifting from a stance of emotional avoidance to one of positive and wishful thinking; this change would represent a transition of adolescent’s emotion-focused coping skills from maladaptive-external to suboptimal-internal [52].

## Limitations

Some limitations must be taken into consideration when interpreting the results of the present study. First, this study focused only on the phenomenological perspective of adolescents and thus did not include the perspectives of other people, such as bullies, other peers, school professionals, and parents or other family members. Indeed, a multi-informant approach would have been particularly useful to explore the intersubjective context of bullying in further detail. A second limitation of our study is the fact that, methodologically speaking, it did not include a psychodynamic approach of discourse analysis [53]. Indeed, as the experience of bullying mobilizes unwanted feelings and attempts to manage those feelings, and since adolescents are frequently not fully aware of the emotional significance of aspects of their experience [54], an approach taking into account their psychological defence mechanisms may have enriched the exploration [53]. Finally, our sampling included heterogeneity in age, sex, and clinical status of adolescents, and further phenomenological studies could be conducted with greater specificity on more homogeneous samples, particularly regarding the presence of post-traumatic stress disorder.

## Implications for clinicians

The results of this study have several practical implications for clinicians working with adolescents in the context of mental health services. First, it appears that a situation of bullying must be sought when an adolescent is seen for unexplained externalized behavioural misconduct. Our results also suggest that low levels of emotional expression in a bullied adolescent may serve as a warning regarding self-harm. Another valuable contribution of these results to practice is the suggestion that a bullied adolescent's tendency to hide this situation from his or her parents may reflect underlying family-related vulnerability, indicating the importance of family support in such cases [55, 56].

The fact that adolescents seemed to benefit from exchanges with people who were capable of understanding emotional experiences related to bullying leads to the recommendation that individual psychological support should be offered to bullied adolescents. This finding may also support the use of therapeutic groups for bullied adolescents, particularly those who are socially isolated in the context of traumatized relationality [56, 57].

## Implications for mental health research

Several new hypotheses arising from the qualitative exploration conducted for this study merit further testing in terms of quantitative work. First, the hypothesis that externalizing behavioural misconduct in adolescents may be associated with covert bullying could be tested via population-based and/or clinically based epidemiological studies. Clinical studies of mental health services could also be conducted to investigate the possible links between a low level of emotional expression and self-harm in bullied adolescents and between the absence of disclosure to parents and family concerns. Finally, the implementation of therapeutic intervention should be evaluated in both contexts.

The study also raises some new questions, which could be the subject of future qualitative studies. In particular, a multi-informant perspective, including the experiences of bullies, other peers, school professionals, and parents or other family members, would be useful to explore the intersubjective context of bullying in further detail. Additionally, other qualitative studies using in-depth focused interviews may improve our understanding of the diversity of the emotional and identity-related consequences of bullying in adolescents.

## Conclusions

This exploratory study shows that emotional suffering and its psychopathological consequences are frequently concealed by bullied adolescents receiving mental health care, and so mental health professionals should be attentive to this possibility to avoiding underestimating such consequents in clinical practice. Phenomenological analysis highlights the potential particularities of the assumptive world of these adolescents and suggests the crucial role played by relationality in their experience. The results suggest incentives to design specific individual and group therapeutic interventions, including family support, for bullied adolescents with significant social withdrawal. Additional research is needed to improve our understanding of the psychopathological and intersubjective aspects of bullying in adolescents.

## Data Availability

The datasets used and/or analysed during the current study are available from the corresponding author on reasonable request.

## Abbreviations

IRB: Institutional review board
COREQ: Consolidated criteria for reporting qualitative research.
FdF: Fondation de France
